# Characterization of the 2009 Pandemic A/Beijing/501/2009 H1N1 Influenza Strain in Human Airway Epithelial Cells and Ferrets

**DOI:** 10.1371/journal.pone.0046184

**Published:** 2012-09-26

**Authors:** Penghui Yang, Jiejie Deng, Chenggang Li, Peirui Zhang, Li Xing, Zhiwei Li, Wei Wang, Yan Zhao, Yiwu Yan, Hongjing Gu, Xin Liu, Zhongpeng Zhao, Shaogeng Zhang, Xiliang Wang, Chengyu Jiang

**Affiliations:** 1 Beijing Institute of Microbiology and Epidemiology, State Key Laboratory of Pathogen and Biosecurity, Beijing, China; 2 State Key Laboratory of Medical Molecular Biology, Institute of Basic Medical Sciences, Peking Union Medical College, Tsinghua University; Chinese Academy of Medical Sciences, Beijing, China; 3 Department of Hepatobiliary, 302 Military Hospital, Beijing, China; University of Liverpool, United Kingdom

## Abstract

**Background:**

A novel 2009 swine-origin influenza A H1N1 virus (S-OIV H1N1) has been transmitted among humans worldwide. However, the pathogenesis of this virus in human airway epithelial cells and mammals is not well understood.

**Methodology/Principal Finding:**

In this study, we showed that a 2009 A (H1N1) influenza virus strain, A/Beijing/501/2009, isolated from a human patient, caused typical influenza-like symptoms including weight loss, fluctuations in body temperature, and pulmonary pathological changes in ferrets. We demonstrated that the human lung adenocarcinoma epithelial cell line A549 was susceptible to infection and that the infected cells underwent apoptosis at 24 h post-infection. In contrast to the seasonal H1N1 influenza virus, the 2009 A (H1N1) influenza virus strain A/Beijing/501/2009 induced more cell death involving caspase-3-dependent apoptosis in A549 cells. Additionally, ferrets infected with the A/Beijing/501/2009 H1N1 virus strain exhibited increased body temperature, greater weight loss, and higher viral titers in the lungs. Therefore, the A/Beijing/501/2009 H1N1 isolate successfully infected the lungs of ferrets and caused more pathological lesions than the seasonal influenza virus. Our findings demonstrate that the difference in virulence of the 2009 pandemic H1N1 influenza virus and the seasonal H1N1 influenza virus *in vitro* and *in vivo* may have been mediated by different mechanisms.

**Conclusion/Significance:**

Our understanding of the pathogenesis of the 2009 A (H1N1) influenza virus infection in both humans and animals is broadened by our findings that apoptotic cell death is involved in the cytopathic effect observed *in vitro* and that the pathological alterations in the lungs of S-OIV H1N1-infected ferrets are much more severe.

## Introduction

In April 2009, an outbreak of influenza in North American was found to be caused by a new swine-origin influenza A (H1N1) virus that has since become prevalent in human populations and has spread worldwide [1], [2], [3]. From June 2009 to August 2010, the world was officially (according to specific World Health Organization [WHO] criteria–WHO phase 6 pandemic alert) in the grip of an influenza A pandemic involving this new strain of the H1N1 virus. Several publications have emphasized the possibility of the reassortment of the 2009 A (H1N1) influenza virus, A/H5N1 viruses or seasonal influenza viruses in humans and the potential serious implications for public health [4], [5].

This 2009 pandemic H1N1 virus can cause human respiratory disease, but its pathogenesis remains poorly understood. In our previous studies, we showed that the S-OIV H1N1 A/Beijing/501/2009 virus replicated in a C57BL/6 mouse model with acute lung injury, and the mice exhibited immune responses mimicking human clinical disease [6]. In addition, we reported that another influenza A (H1N1) virus strain, A/Wenshan/01/2009 H1N1, isolated in the Yunnan Province of China induced significant apoptotic cell death in the human lung adenocarcinoma epithelial cell line A549 [7]. In this report, we investigated the infection and pathogenesis of this new 2009 pandemic strain, A/Beijing/501/2009 H1N1, in A549 cells, compared to the A/California/07/2009 H1N1 virus and the contemporary seasonal H1N1 influenza virus.

Previous animal studies have indicated that the 2009 pandemic H1N1 is slightly more pathogenic than the contemporary seasonal H1N1 viruses [1], [8]. Ferrets (*Mustela putorius furo*) are a suitable animal model for studying human influenza virus infections because they are susceptible to natural infection and can develop respiratory disease and lung pathology similar to humans with influenza virus infections [9], [10], [11]. A ferret model was used to compare the clinical manifestations in ferrets infected with the A/Beijing/501/2009 influenza H1N1 strain, the A/California/07/2009 H1N1 strain and seasonal H1N1 influenza strain and to determine whether the 2009 pandemic H1N1 virus displays stronger pathogenesis in the respiratory system.

## Materials and Methods

### Ethics Statement

All procedures were conducted under protocols approved by the Institute of Animal Care and Use Committee (ID: SYXK 2007-005) at AMMS, all facilities were accredited by the AMMS Animal Care and Ethics Committee, and guidelines for ferret housing, environment and comfort described in the Guide For The Care and Use of Laboratory Animals, National Research Council, were strictly adhered to. All infections and sample collections were performed under 5% isoflurane anaesthesia and all efforts were made to minimize suffering.

Subject provided written informed consent for participation in the study.

### Case Reports

Patient: A 69-year-old male entered the 302 Military Hospital on 22 May 2009 with a high fever (37.9°C) that started 15 hours prior. He complained of diffuse pain predominantly in the lower abdomen, nausea, vomiting, runny nose, sore throat, coughing, weakness and fever blisters on his lips. A 5-ml sample of a saline wash of his throat was sent to the laboratory.

### Isolation of Virus

A 0.2-ml volume of the throat wash was inoculated into the allantoic cavities of 10-day-old SPF embryonated eggs and were incubated at 34°C for 72 h. The first two passages (three and five eggs) were negative, but on the third passage, three of five eggs showed hemagglutination titers of 1∶16, 1∶4, and 1∶32, respectively. Blind passages were performed using samples from a pool of infected allantoic fluids. MDCK cells (Madin-Darby canine kidney cells) were simultaneously infected with virus from the eggs, and positive results were confirmed by the hemagglutination of supernatants and a hemadsorption assay at 4°C. The virus titers were 10^3.5^ TCID_50_ (50% tissue culture infective doses) in the presence of trypsin (1 µg/ml) and 10^1.5^ TCID_50_ without trypsin. The virus strain was verified by the inhibition assay using anti-sera to A/California/07/2009 (1∶320) and A/Sichuan/01/2009 (1∶320).

### Viruses and Cell Lines

The influenza A H1N1 virus A/Beijing/501/2009 (BJ501) was isolated from a patient from Beijing in 2009 [5], [12], [13]. The genomic sequences are available in GenBank under the accession numbers GQ223408-GQ223415. The wild-type H1N1 influenza virus A/California/07/2009 virus (CA07) used in this study was kindly provided by the Influenza Branch of the Center for Disease Prevention and Control of China [4], [14], [15], [16], [17], [18], [19]. Virus stocks were propagated in specific-pathogen free (SPF) chicken embryos (Laboratory Animal Center, Beijing, China). Diluted virus was injected into the allantoic cavity of 10-day-old SPF chicken eggs and incubated at 34°C. Allantoic fluids were harvested 48 h after inoculation. Infectious allantoic fluids were pooled and stored at −80°C until use. The 50% tissue culture infectious dose (TCID_50_) for each virus was determined by serial dilution of the virus in Madin-Darby canine kidney (MDCK) cells (ATCC, Virginia, USA) and calculated by the method developed by Reed and Muench [20]. All experiments with the wild-type virus A/Beijing/501/2009 and A/California/07/2009 were performed in the bio-safety level 3 animal facilities approved by the AMMS.

The human lung adenocarcinoma epithelial cell line A549 was purchased from ATCC and cultured in DMEM (Hyclone) supplemented with 10% FBS (Hyclone) and 100 U/ml penicillin/streptomycin at 37°C with 5% CO_2_. The primary antibodies anti-PARP and anti-caspase 3 were purchased from Cell Signaling Technology Company. The anti-β-actin antibody was purchased from Sigma-Aldrich.

### MTT Assay

Cells were seeded in 96-well plates at a density of 1×10^5^ cells/ml. Influenza virus or allantoic fluid was added to the wells the next day. Each group was represented by triplicate wells. After incubating the samples for the indicated times, 20 µl of CellTiter 96 Aqueous One Solution Cell Proliferation Assay (Promega) was added to each well, and the samples were incubated at 37°C for another 2 h. The absorbance was then measured at 490 nm.

### Western Blotting

Cells were seeded at 1×10^5^ cells/ml in 12-well plates. H1N1 virus or an equal volume of allantoic fluid was added to the wells the next day, and the cells were incubated for another 24 h. Cells were lysed in lysis buffer (RIPA lysis buffer containing protease inhibitors), and proteins were denatured at 97°C for 10 min and analyzed by western blot.

### TUNEL Assay

Cells were seeded at a density of 1×10^5^ cells/ml on cover slips in 24-well plates. One day later, virus infection was performed at an MOI of 3.0, and cells were incubated for another 24 h. Apoptotic cells were characterized by positive terminal deoxynucleotidyltransferase-mediated dUTP-biotin nick end labeling (TUNEL) staining following the manufacturer’s instructions (In Situ Cell Death Detection Kit, POD; Roche). Briefly, cells were fixed with paraformaldehyde in PBS and permeabilized with 0.1% Triton X-100. The nonspecific endogenous peroxidases were inactivated with 3% hydrogen peroxide in methanol. After the TdT reaction, dUTPs were visualized using HRP-conjugated antibody and 3,3′-diaminobenzidine (DAB).

TUNEL staining was also performed on the paraffin-embedded sections of ferret lung tissue. In short, after deparaffinization and rehydration, sections were digested with proteinase K at a concentration of 20 µg/ml for 15 minutes. The nonspecific endogenous peroxidases were quenched with 3% hydrogen peroxide in methanol. After the TdT reaction, dUTPs were visualized using HRP-conjugated antibody and DAB. The slides were counterstained with haematoxylin. 100 random lung fields per group were captured at a 400× magnification and the percentage of TUNEL positive area was calculated by the Image Plus software.

#### RNA interference

SiRNA against caspase-3 were purchased from Sangon Biotech (Shanghai) Co., Ltd. siRNA-1, UGGAUUAUCCUGAGAUGGGTT (nucleotides [nt’s] 351 to 369), and siRNA-2, AGUGAAGCAAAUCAGAAACTT (nt’s 2148 to 2166) [21]. A549 cells were transfected with the siRNA (50 nm) according to the manufacturer’s instruction and the MTT assay was performed as descibed above.

### Experimental Infection of Ferrets

We used 10–12 week old female ferrets (Angora LTD, Jiangsu, China) that were tested negative for circulating influenza virus by the serological hemagglutination inhibition (HI) assay. The baseline body temperatures and body weights were measured prior to infection. Nine ferrets per group were anaesthetized by intramuscular injection of ketamine and xylazine (5 mg per kg of body weight and 0.5 mg per kg of body weight, respectively) and intranasally inoculated with 10^7^ TCID_50_ (200 µl) of the BJ501, CA07 or seasonal H1N1 viruses, respectively. At days 3 and 7 post-infection, 3 ferrets per group were euthanized and the nasal turbinates, lungs, spleen, kidney, liver and brain were harvested for virological and pathological examination. In the virological examination, tissues were homogenized in MEM medium containing antibiotics to make a 10% w/v tissue homogenates for both the day 3 and day 7 post-infection groups. The supernatant of tissue homogenates obtained after low-speed centrifugation were titrated into 24- and 96-well culture plates containing MDCK cells, and the titers are expressed as log_10_TCID_50_/g tissue [22].

### Histopathological Analysis

The lung tissues of infected ferrets were harvested and immediately fixed in 4% neutral formalin for 48 h and then embedded in paraffin. Ultrathin sections were obtained and stained with hematoxylin-eosin, and the histopathological alterations were determined using light microscopy. The number of infiltrating cells was counted in 100 microscopic fields for each group at a magnification of 1000 X.

### Statistical Analyses

All data are shown as the means ± S.E.M. Measurements at single time points were analyzed using an ANOVA, and if they demonstrated significance, they were further analyzed by a two-tailed t-test. p<0.05 indicates statistical significance.

## Results

### The A/Beijing/501 H1N1 Virus Strain Induces Cell Death in A549 Cells

**Figure 1:**
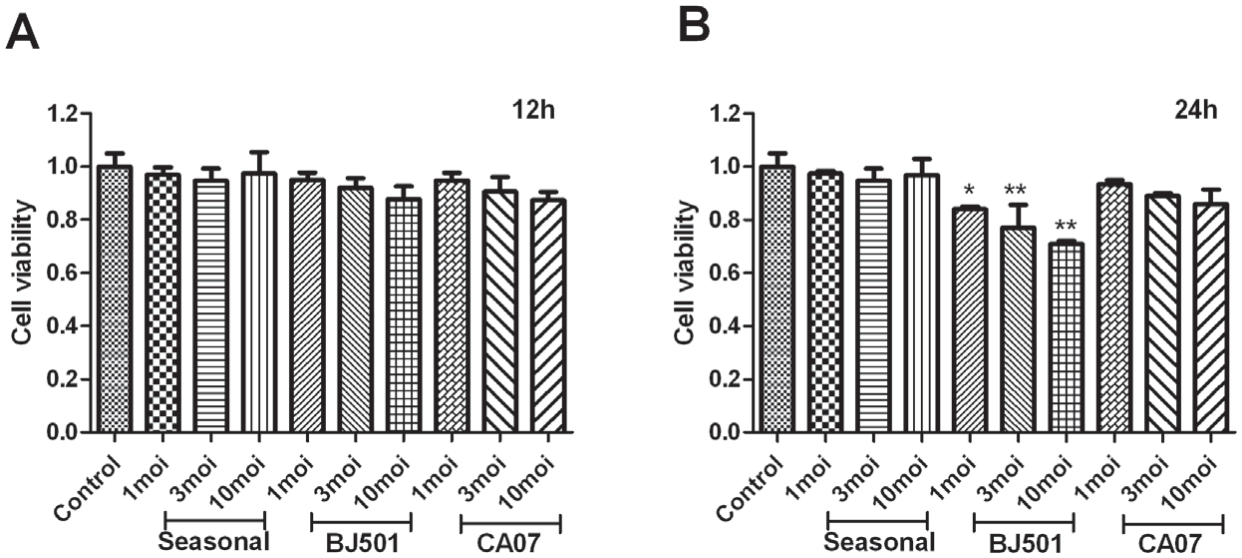
A/Beijing/501 H1N1-induced cell death in the human lung adenocarcinoma epithelial cell line A549. MTT assay of A549 cells treated with allantoic fluid, seasonal H1N1 virus, A/Beijing/501 H1N1 virus and A/CA07 H1N1virus at 12 hours (A) and 24 hours (B) post infection. *p<0.01, **p<0.001 versus the control.

A549 cells were infected with A/Beijing/501 H1N1, a virus strain that emerged in Beijing, and the resulting cytotoxicity was compared with the toxicity induced by the A/California/07/2009 H1N1 virus strain and a seasonal H1N1 strain. We used the human lung adenocarcinoma epithelial cell line A549 because these cells originate from the pulmonary alveoli and are sensitive to virus infection. The cells were infected with the three H1N1 strains at multiplicities of infection (MOIs) of 1, 3, and 10, and the cells were incubated at 37°C. The cell viability was measured using the MTT assay at 12 and 24 h post-infection. The cell viability did not decrease among any of the influenza H1N1-infected A549 cells at 12 h post-infection (Figure 1A); however, we discovered that the infection with the A/Beijing/501 strain (MOI 1, MOI 3 and MOI 10) induced significant cell death at 24 h post-infection. The cell viability decreased gradually in A/Beijing/501 H1N1-infected A549 cells in a dose-dependent manner (Figure 1B). This finding suggests that the infection with 2009 pandemic A/Beijing/501 H1N1 virus can induce progressive and irreversible cytotoxicity in A549 cells, whereas infection with the seasonal H1N1 virus does not induce significant cytotoxicity at the early stages of infection. Meanwhile, in A549 cells, we found A/Beijing/501 H1N1 virus exhibited high replication rate from 3 h post-infection; in contrast, CA07 virus did not produce significant levels of viral titers (Figure S1). On the basis of these results, we speculated that there are different infection response mechanisms in the human respiratory tract for 2009 pandemic H1N1 viruses isolated from different regions.

### Caspase-3-dependent Apoptosis is Involved in A/Beijing/501 H1N1-induced A549 Cell Death

**Figure 2:**
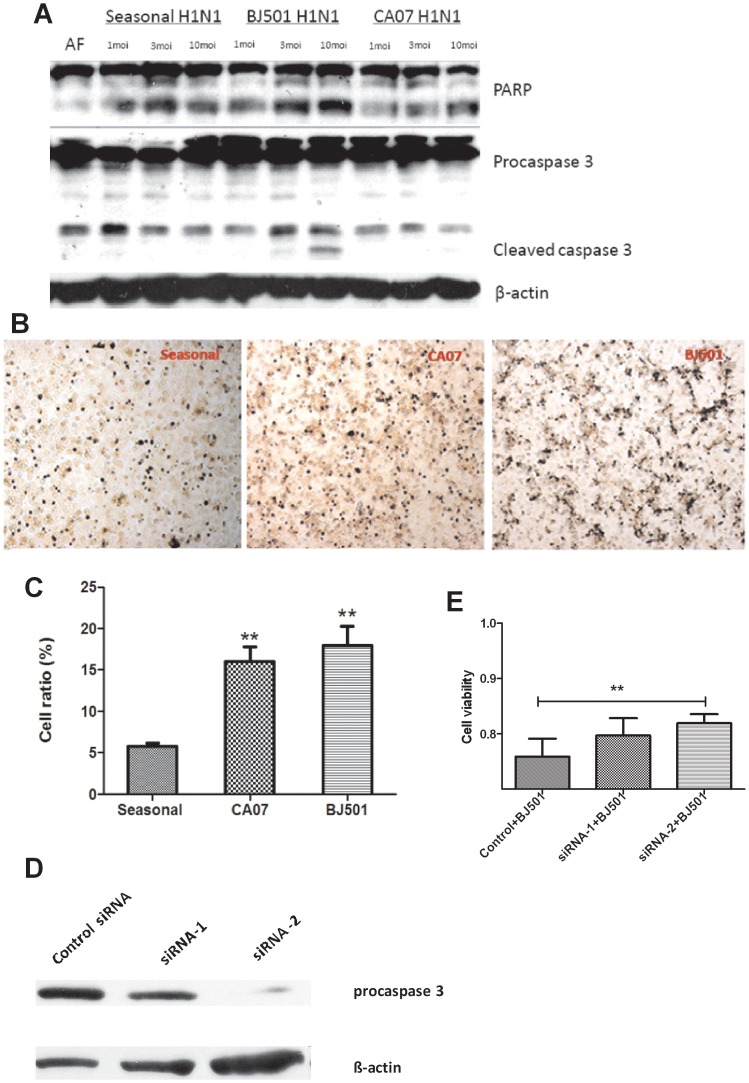
A/Beijing/501 induces apoptosis in the A549 cells. (A) Western blot analysis of mock-infected and H1N1-infected A549 cell lysate with anti-caspase 3, anti-PARP and anti-β-actin antibodies after a 24 h infection. (B–C) Cells were infected with seasonal H1N1, A/Beijing/501 H1N1 and A/CA/07 H1N1 influenza viruses. In situ apoptosis was detected using a FITC-dUTP labeled TUNEL assay and statistical analysis of relative proportion of TUNEL positive cells. For quantification, >1000 cells were scored in three independent experiments. (D–E) Knockdown of caspase 3 inhibits A/Beijing/501 H1N1 virus replication. A549 cells were transfected with the control, siRNA-1 and siRNA-2 against caspase 3 (50 nm). 24 hours later, Western blot analysis (D) and MTT assay (E) of A/Beijing/501 H1N1-infected A549 cells. **p<0.001.

To elucidate the type of cell death induced during A/Beijing/501 H1N1 virus infection, we investigated whether A/Beijing/501 H1N1 virus infection induces apoptotic cell death. First, we analyzed hallmarks of apoptosis, PARP and caspase-3 activation, by immunoblot analysis. Twenty-four hours after infection, activated caspase-3 was only detected in A549 cells infected with A/Beijing/501 H1N1 virus at MOI 10 but not in the mock-infected control, seasonal H1N1-infected cells or A/California07 H1N1-infected cells. PARP, a death substrate that is required for caspase-3 activation, was detected as an 89-kDa, proteolytically cleaved fragment in all three H1N1 influenza virus-infected cells. With increasing MOIs, the PARP cleaved protein bands gradually increased in the A/Beijing/501 H1N1-infected groups. These results suggest that apoptosis contributed to the A/Beijing/501 H1N1-induced cell death, and the cell death was caspase-3 dependent (Figure 2).

To further confirm our observation, we performed the TUNEL assay, which uses terminal deoxynucleotidyltransferase (TdT) to catalyze the addition of FITC-labeled dUTPs onto nicks in DNA [7]. As shown in Figure 2B, the proportions of apoptotic cells observed in A/Beijing/501- and A/California/07 H1N1-infected cells (the black dots) were much greater than in seasonal H1N1-infected cells at 24 h post-infection. Statistical analysis also revealed that the percentage of apoptotic cells was significantly higher in A/Beijing/501 H1N1- and A/California/07 H1N1-infected cells than in seasonal H1N1-infected cells (Figure 2C). In addition, when caspase-3 siRNA were used in the A/Beijing/501 H1N1-infected A549 cells, western blot analysis and MTT assay results showed that caspase-3 siRNA could significantly inhibit A/Beijing/501 H1N1 virus replication (Figure 2D and 2E).

### Pathogenesis and Virulence of the Different Viral Isolates in Ferrets

**Figure 3:**
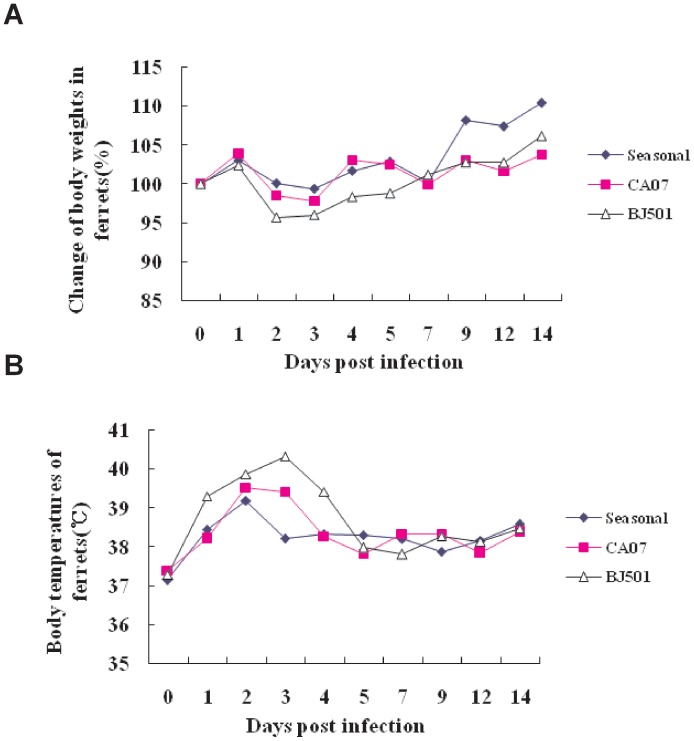
Body weight alterations (A) and body temperature fluctuations (B) in ferrets inoculated with seasonal H1N1, A/Beijing/501 H1N1 and A/CA07 H1N1 influenza virus. Three groups of nine ferrets were individually inoculated with 10^7^ TCID_50_ of the seasonal H1N1 or the 2009 pandemic H1N1 virus intranasally. The body weights and the rectal body temperatures of infected ferrets were recorded for up to 14 days post-infection.

To assess the virulence of the A/Beijing/501 H1N1 strain in ferrets, nine female ferrets separated into three groups were inoculated intranasally with 10^7^ TCID_50_ of virus. The animals were monitored for clinical signs and were weighed daily as an indicator of the disease. Both the A/Beijing/501 H1N1 and the A/California/07 H1N1 viruses caused sneezing, ruffled fur, decreased appetite, and nasal discharge in the ferrets. The mean maximum weight loss was 10% for animals inoculated with the A/Beijing/501 H1N1 influenza virus at two days post-infection. The body weight of the ferrets infected with any of the 2009 A (H1N1) influenza viruses gradually returned to the original weight by 7 days post-infection. On the contrary, there was no significant weight loss observed in the seasonal H1N1 infected-ferrets (Fig. 3A). All of the animals rapidly developed a fever that peaked at day 2–3 post-infection, and the body temperature remained elevated for 2 days and returned to normal at 5 days post-infection (Fig. 3B).

To evaluate the replication of the viruses in different tissues, three animals of each group were sacrificed at days 3 and 7 post-infection. Sections of the nasal turbinates, trachea, lungs, liver, spleen, kidney, and brain tissue were homogenized and used for the determination of virus titers by means of endpoint titration in MDCK cells. Among the tissue types, the virus replicated efficiently in the upper and lower respiratory tract of virus infected-ferrets and reached detectable titers at 3 days post-infection. The seasonal and 2009 A (H1N1) influenza viruses were both detectable in the nasal turbinates of inoculated ferrets at 3 days after inoculation, whereas the A/Beijing/501 H1N1 influenza virus- and A/California/07 H1N1 influenza virus-infected group yielded slightly higher virus titers (10^6.8^ versus 10^6.6^ TCID_50_ per gram of tissue, respectively) (Table 1). The A/Beijing/501 H1N1 and A/California/07 H1N1 strains were also detected in the trachea (10^3.85^ versus 10^4.0^ TCID_50_ per gram of tissue) and lungs (10^4.6^ versus 10^4.3^ TCID_50_ per gram of tissue) of inoculated ferrets, whereas the seasonal influenza virus was not detectable in the lungs. Seven days post-inoculation, all virus strains were detected at relatively low titers in the nasal turbinates, trachea and lungs. Interestingly, the A/Beijing/501 H1N1 strain was detected in brains of the animals (10^1.25^ TCID_50_ per gram of tissue) at 3 days post-infection, and the virus was below the detection limit 7 days post-inoculation. No virus was detected in the liver, spleen, or kidneys of animals inoculated with any of the viruses at 3 or 7 days after inoculation. These results show that the 2009 A (H1N1) influenza virus can be cleared from the respiratory tract within 7 days post-infection.

**Table 1:** Replication of 2009 A(H1N1) influenza viruses and a seasonal H1N1 virus in ferrets^a^.

Tissue	Virus titer (LogTCID_50_/gram tissue)					
	Day 3 after inoculation			Day 7 after inoculation		
	Seasonal A(H1N1)	A/Beijing501	A/California07	Seasonal A(H1N1)	A/Beijing501	A/California07
Nasal turbinates	5.25±0.10	6.80±0.10	6.60±0.10	3.30±0.15	2.80±0.10	2.55±0.02
Trachea	≤1.0^b^	3.85±0.05	4.00±0.36	≤1.0^b^	2.22±0.18	2.00±0.12
Lung	≤1.0^b^	4.60±0.12	4.30±0.62	≤1.0^b^	1.62±0.15	1.80±0.10
Brains	≤1.0^b^	1.25±0.10	≤1.0^b^	≤1.0^b^	≤1.0^b^	≤1.0^b^

a Three ferrets were inoculated i.n. with 10^7^ TCID_50_ of the indicated virus, respectively. These animals were euthanized at 3 and 7 days after inoculation. Virus titers in nasal turbinates, trachea, lungs and brain tissue were determined by means of end-point titration in MDCK cells. No virus was detected in liver, spleen, and kidney tissue for either virus and was thus not included in the table. Geometric mean titer ± SD is indicated.

b Lower limit of detection is 10TCID_50_/g of tissue.

We further analyzed caspase 3 and PARP activation in the lung tissues of ferrets by immunoblot analysis. At 3 dpi, activated caspase-3 was detected in A/Beijing501 H1N1-infected lung tissues of ferrets, but not in the CA07 H1N1-infected lung tissues (Figure S2A). And TUNEL assay was performed in the lung tissues of ferrets, and apoptotic cells were clearly observed in A/Beijing501 and CA07 H1N1-infected lung tissues, but very few in seasonal H1N1-infected tissues at 3 and 7 dpi (Figure S2B). Finally, we analyzed the percentage of TUNEL-positive areas in A/Beijing501, CA07 and seasonal H1N1-infected lung tissues of ferrets (Figure S2C), but few TUNEL-positive cells were detected in seasonal H1N1-infected tissues.

### The A/Beijing/501 H1N1 Virus Causes more Sustained and Widespread Lung Damage in Ferrets

**Figure 4:**
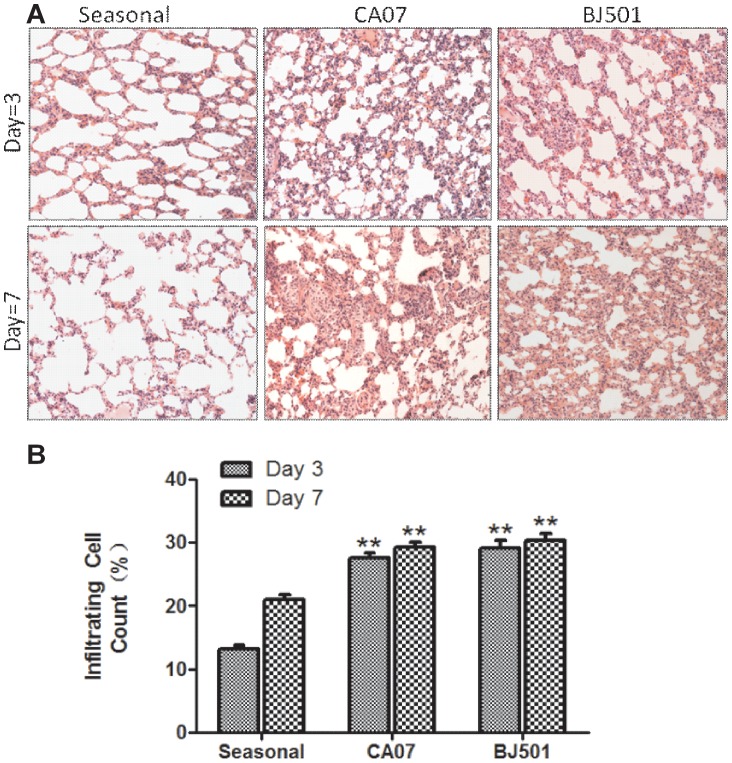
Histopathological examination of lung tissue. Representative H&E staining of lung sections from ferrets inoculated with seasonal or 2009 A(H1N1) influenza virus at 3 and 7 days post-infection, respectively. (A) Magnification = 200X. (B) The mean number of infiltrating cells per microscopic field ± SEM are shown. Statistical analysis of the infiltrating cell number differences between the seasonal influenza-infected groups and the 2009 A (H1N1) influenza-infected groups (n = 100 fields analyzed for three ferrets per group).*p<0.01, **p<0.001.

As reported previously, the swine-origin 2009 A (H1N1) viruses cause more severe pathological lung damage than the seasonal strains [4]. In our study, at 3 and 7 days post-inoculation, three animals from each group were independently euthanized, and the nasal turbinates, trachea, lungs, liver, spleen, kidney, and brains were harvested for pathological and virological examination. At 3 days post-inoculation, gross examination of the lungs revealed focal to multifocal mild lesions in all ferrets from each group. Lung histopathological analyses revealed that ferrets inoculated with the A/Beijing/501 H1N1 or A/California07 H1N1 influenza virus had more severe pulmonary inflammation and large quantities of infiltrating cells compared to the ferrets inoculated with seasonal influenza virus at 3 or 7 days post-inoculation (Fig. 4A). Statistical analysis showed that the mean numbers of infiltrating cells per microscopic field were significantly higher in the A/Beijing/501 H1N1 and A/California/07 H1N1-infected lung of ferrets than in the seasonal H1N1 influenza infected group (Fig. 4B). Although the influenza H1N1 isolates caused similar histopathological changes at early disease stages, the damage was more widespread in the case of A/Beijing/501 H1N1 strain.

## Discussion

Humans infected with the 2009 pandemic H1N1 suffer from fever, cough, sore throat, diarrhea, and vomiting [23]. Despite their overall genetic similarity, early H1N1 strains vary considerably in their virulence based on the region of isolation and the animal models employed [4], [24]. We investigated the pathogenesis of the 2009 pandemic A/Beijing/501 H1N1 strain isolated in Beijing in human airway epithelial cells and ferrets. In agreement with our previous studies, we found that the A/Beijing/501 strain can cause pronounced cytopathic effects in the human lung epithelial cell line A549, suggesting that the alveolar epithelial cell may also be a target at an early stage of infection with the 2009 pandemic virus. The cell death in A549 cells was attributed to virus-induced caspase-3-dependent apoptosis. Based on these results, we speculate that the apoptotic cell death induced by the A/Beijing/501 H1N1 virus and other homologous strains in airway epithelial cells may be responsible for the critical illness experienced by some patients.

Our study using a ferret model has demonstrated that the 2009 pandemic H1N1 strain causes more severe clinical signs and symptoms (e.g., elevated body temperature and reduced body weight) and produces higher viral titers than does the seasonal H1N1 influenza virus.

Programmed cell death, or apoptosis plays an important role in the pathogenesis of many infectious disease, including those caused by virus [7]. Influenza virus induces apoptosis in many cell types both in vivo and in vitro, to some extent, apoptosis is a protective response of host cell to virus infection [8], [24], [25], [26]. Our previous studies have firstly confirmed that the 2009 pandemic H1N1 A/Wenshan/01/2009 strain could induce apoptosis in human epithelial cell lines and the viral entry was mediated by clathirin-and dynamin-dependent endocytosis in CNE-2Z and A549 cell lines [7]. Here, we demonstrated that the 2009 pandemic H1N1 A/Beijing/501/2009 could induce caspase-3-dependent apoptosis in A549 cells and ferrets, which is likely to contribute to virus pathogenesis.

In the 2009 pandemic H1N1-infected groups, weight loss was more prominent, and the animals required a longer recovery period, which may have resulted from the tissue damage caused by profound cytokine production and broader tissue tropism of pandemic strains as reported by others in this field [4], [27], [28]. In addition, the sustained fever may have affected the animal’s appetite because body weight recovery did not occur until the body temperature returned to baseline. Consistent with other H1N1 studies in ferrets [29], [30], we detected viral titers in lung from both the 2009 A/Beijing/501 H1N1- and the A/California/07 H1N1-infected groups at day 3 and 7 post-infection. Ferrets infected with A/Beijing/501 H1N1 virus strain exhibited mild clinical signs of infection. The virus replicated efficiently in the lungs and to a lesser extent in the brain; however, there was no detectable replication in any other organs. In the seasonal H1N1 influenza virus-infected ferrets, the level of replication was below the detection limit in the lungs. Our results are in accordance with earlier studies using ferret, mouse and monkey animal models [27], [29], [31]. Seasonal H1N1 influenza virus replication can only be detected in the nasal turbinates, but 2009 pandemic H1N1 replication can be detected in nasal turbinates, trachea, lungs, and even in the brain of the animals at 3 days post-inoculation. Our findings indicate that the 2009 A (H1N1) influenza virus replicates efficiently in the upper and lower respiratory tract of ferrets. These results are in agreement with observations from influenza virus infection in humans, where mild symptoms are common, but relatively efficient human-to-human transmission has been observed.

In conclusion, the more severe pathological signs in some humans or animals infected with the 2009 pandemic H1N1influenza virus compared to the seasonal H1N1 influenza virus appear to be attributed to the severe apoptotic cell death in human airway epithelial cells and the ability of the virus to replicate in the lungs of infected ferrets.

## Supporting Information

Figure S1 The growth kinetics of seasonal H1N1, A/Beijing/501 H1N1 and A/CA/07 H1N1 virus in A549 cells. A549 cells were inoculated with 10 MOI of virus. At the indicated times, cells were collected and virus titers were determined by TCID_50_ in MDCK cells.
(TIF)

Figure S2 A/Beijing/501 induces apoptosis in the lung tissue of ferrets. (A)Western blot analysis of CA07 and BJ501 H1N1-infected lung tissue of ferrets with anti-caspase 3, anti-PARP and anti-β-actin antibodies at 3 dpi. (B–C) Ferrets were infected with seasonal H1N1, A/Beijing/501 H1N1 and A/CA/07 H1N1 influenza viruses. At 3 and 7 days post-infection, the lung tissues of ferrets were examined by TUNEL assay and statistical analysis of relative proportion of TUNEL positive cells. For quantification, 100 random lung fields per group were captured at a 400× magnification and the percentage of TUNEL positive area was calculated by the Image Plus software. **p<0.001
(TIF)
