# Supplementary figures and images for: Characterization of the 2009 Pandemic A/Beijing/501/2009 H1N1 Influenza Strain in Human Airway Epithelial Cells and Ferrets
Source: PLoS One. 2012 Sep 26;7(9):e46184. doi: 10.1371/journal.pone.0046184 (PMC3458874; doi:10.1371/journal.pone.0046184)

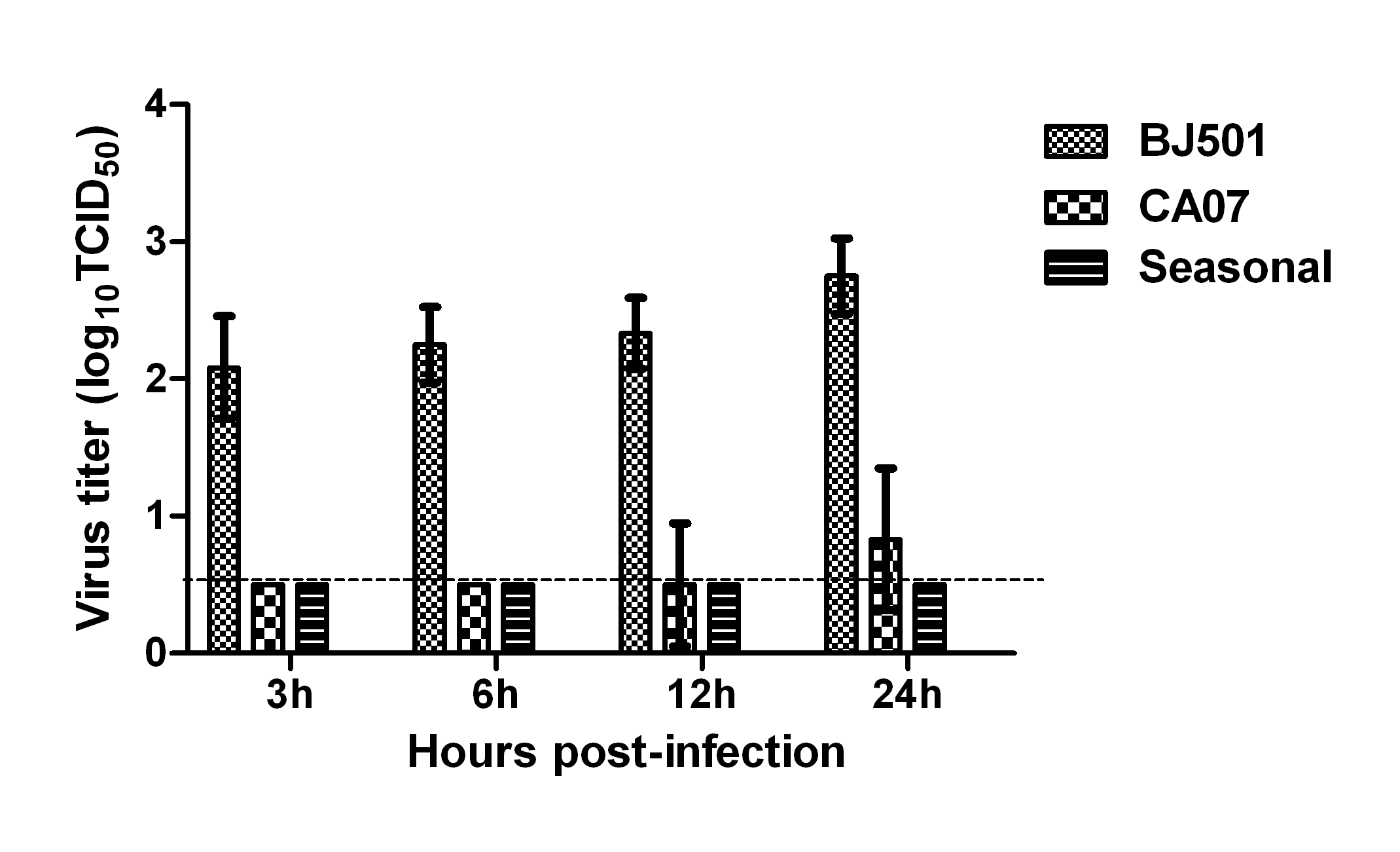

Supplement: Figure S1 — The growth kinetics of seasonal H1N1, A/Beijing/501 H1N1 and A/CA/07 H1N1 virus in A549 cells. A549 cells were inoculated with 10 MOI of virus. At the indicated times, cells were collected and virus titers were determined by TCID50 in MDCK cells. (TIF) [file pone.0046184.s001.tif]

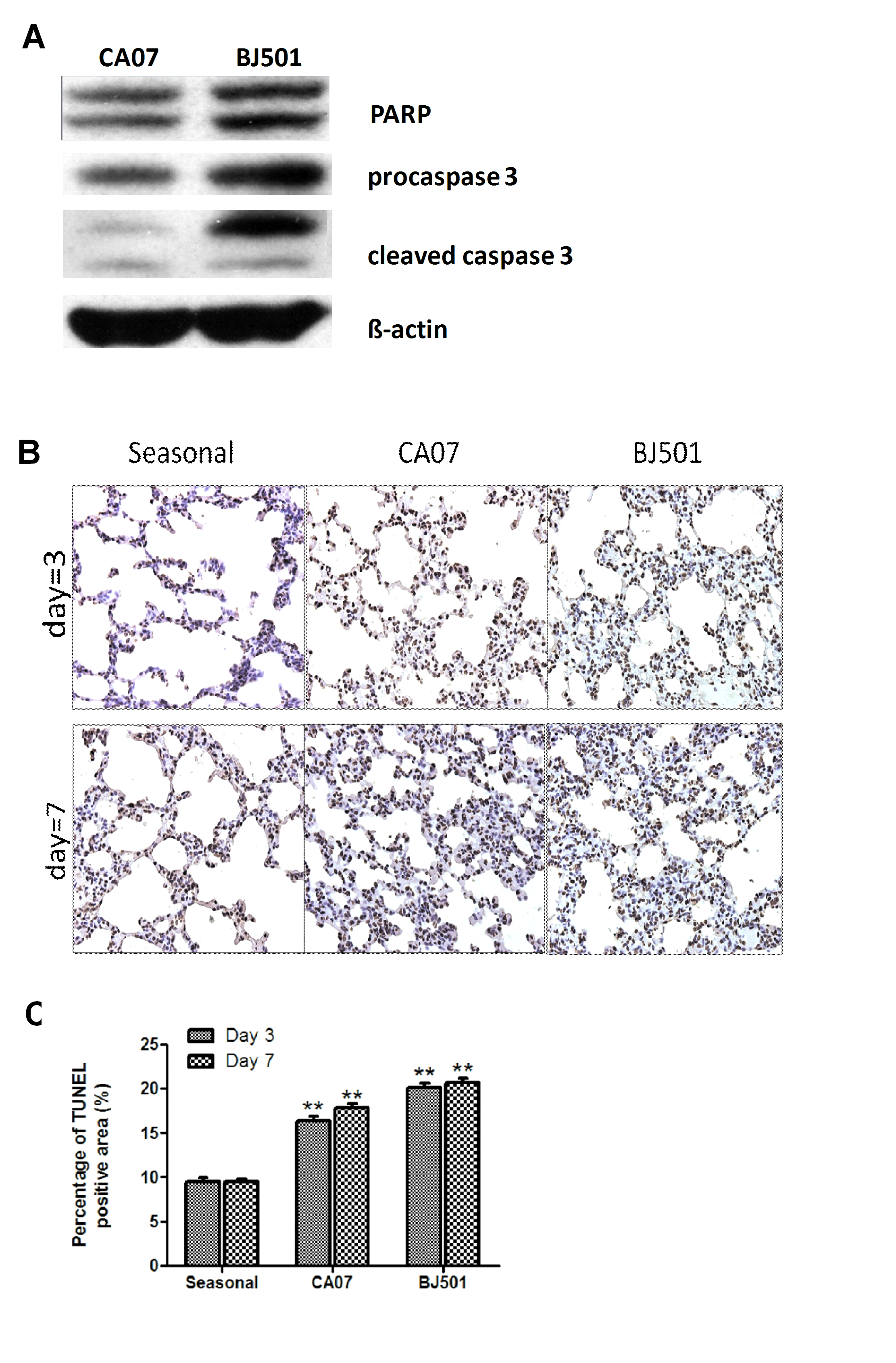

Supplement: Figure S2 — A/Beijing/501 induces apoptosis in the lung tissue of ferrets. (A)Western blot analysis of CA07 and BJ501 H1N1-infected lung tissue of ferrets with anti-caspase 3, anti-PARP and anti-β-actin antibodies at 3 dpi. (B–C) Ferrets were infected with seasonal H1N1, A/Beijing/501 H1N1 and A/CA/07 H1N1 influenza viruses. At 3 and 7 days post-infection, the lung tissues of ferrets were examined by TUNEL assay and statistical analysis of relative proportion of TUNEL positive cells. For quantification, 100 random lung fields per group were captured at a 400× magnification and the percentage of TUNEL positive area was calculated by the Image Plus software. **p<0.001 (TIF) [file pone.0046184.s002.tif]
